# Combining full-length transcriptome sequencing and next generation sequencing to provide insight into the growth superiority of the hybrid grouper (*Cromileptes altivelas* (♀) × *Epinephelus lanceolatus* (♂))

**DOI:** 10.1371/journal.pone.0308802

**Published:** 2024-10-09

**Authors:** Liu Cao, Jun Ma, Yan Lu, Pan Chen, Xingrong Hou, Ning Yang, Hai Huang

**Affiliations:** 1 Yazhou Bay Innovation Institute, Hainan Tropical Ocean University, Sanya, China; 2 Hainan Key Laboratory for Conservation and Utilization of Tropical Marine Fishery Resources, Hainan Tropical Ocean University, Sanya, China; 3 Key Laboratory of Utilization and Conservation for Tropical Marine Bioresources of Ministry of Education, Hainan Tropical Ocean University, Sanya, China; 4 College of Fisheries and Life Sciences, Hainan Tropical Ocean University, Sanya, China; Sher-e-Kashmir University of Agricultural Sciences and Technology of Kashmir, INDIA

## Abstract

The hybrid grouper (*Cromileptes altivelas*, ♀ × *Epinephelus lanceolatus*, ♂) is an economically important aquaculture species that exhibits certain growth advantages compared to its female parent, *Cromileptes altivelas*. However, the current understanding of the molecular mechanisms underlying the growth of hybrid groupers is lacking. Herein, we performed full-length transcriptome sequencing and next-generation sequencing on the hybrid grouper and its parents to identify growth-related genes and comprehensively analyze the regulatory mechanism of growth heterosis in the hybrid grouper. Approximately 44.70, 40.44, and 45.32 Gb of single-molecule real-time sequencing data were generated in *C*. *altivelas* (Cal), *E*. *lanceolatus* (Ela), and the hybrid (Hyb), which were combined into 204,322 non-redundant isoforms using the PacBio sequencing platform. Differentially expressed genes (DEGs) were identified between Hyb and Cal (3,494, 2,125, and 1,487 in brain, liver, and muscle tissues, respectively) and Hyb and Ela (3,415, 2,351, and 1,675 in brain, liver, and muscle tissues, respectively). Then, 27 DEGs (13 in the brain and 14 in the muscle) related to growth traits were identified using cluster and correlation network analysis. Quantitative RT-PCR validated 15 DEGs consistent with transcriptome sequencing (RNA-seq) trends. The Kyoto Encyclopedia of Genes and Genomes (KEGG) pathway analysis revealed that these 15 genes were mainly involved in regulating the actin cytoskeleton, suggesting that this pathway plays an essential role in fish growth. In addition, we found that the phosphatase and tensin homologue (PTEN) is a key regulator of growth heterosis in Hyb. These results shed light on the regulatory mechanism of growth in the Hyb, which is important for marker-assisted selection programs to improve the growth quality of groupers.

## Introduction

Growth, a pivotal economic aspect of aquaculture, profoundly influences profitability and productivity. Its intricate regulation has been extensively investigated in various aquatic species, including *Pangasianodon hypophthalmus* [1], *Trachinotus ovatus* [2], *Lateolabrax maculatus* [3] and *Epinephelus coioides* [4]. Growth dynamics are governed by intricate signaling pathways originating from multiple tissues. Among these, the growth hormone/insulin-like growth factor (GH/IGF) system stands out as a principal promoter of growth in vertebrates [5]. Growth hormone (GH) orchestrates somatic growth by binding to GH receptors [6,7]. Structurally akin to insulin, insulin-like growth factors (IGFs), including IGF1 and IGF2, stimulate myogenic cell proliferation, differentiation, and protein synthesis [8,9]. GH/IGF system mediates its biological functions via signaling pathways such as mitogen-activated protein kinase/extracellular signal-regulated kinase (MAPK/ERK), phosphatidylinositol 3-kinase/protein kinase B/Forkhead box O (PI3K/Akt/FOXO), and JAK2-STAT5 [10–12]. Expression of growth-related genes within the GH/IGF axis predominantly occurs in brain, liver, and muscle tissues [13,14].

Body weight serves as a pivotal metric for assessing the growth rate of aquatic species, with muscle mass often constituting 50%-70% of total body weight [15]. Hence, enhancing muscle growth is crucial for augmenting the yield of cultured fish. Research underscores the indispensable role of high-quality muscle contractions in muscle growth, a process intricately intertwined with actin cytoskeleton dynamics [16,17]. Mounting evidence suggests that smooth muscle contraction necessitates actin filament polymerization and various cytoskeletal processes [18].

Groupers are esteemed for their economic significance in marine aquaculture with production volume of over 200,000 tons in 2022 [19]. However, the protogynous hermaphroditism characteristic of groupers poses challenges for attaining the F2 generation via self-crossing [20]. Hybridization emerges as a valuable strategy in grouper mariculture, yielding numerous farmed species, including *Epinephelus fuscoguttatus* (♀) × *Epinephelus polyphekadion* (♂) [21], *E*. *awoara* (♀) × *E*. *tukula* (♂) [22], *E*. *fuscoguttatus* ♀ × *E*. *lanceolatus* ♂ [23] and *Cromileptes altivelas* (Cal, ♀) × *E*. *lanceolatus* (Ela, ♂) [24]. *C*. *altivelis* × *E*. *lanceolatus* (Hyb) are important mariculture species in the southern coastal region of China. Previous research has demonstrated the superior growth performance of Hyb over Cal [25]. However, the mechanisms underlying the growth superiority of Hyb have not yet been reported.

Transcriptome sequencing (RNA-seq) is a high-throughput, low-cost technology that utilizes next-generation sequencing to reveal the presence and quantity of RNA in a sample at a given moment. This technique is most widely applied in analyzing the regulatory mechanisms of various important economic traits in aquatic animals [26]. Regulatory factors and mechanisms associated with growth and skin pigmentation in *E*. *fuscoguttatus* (♀) × *E*. *lanceolatus* (♂), as well as body pigmentation formation in *Plectropomus leopardus*, have been discovered by next-generation sequencing technology [12,27,28]. However, next-generation sequencing technology has apparent disadvantages, such as the short length of sequencing reads, unreliable assembly results, and limited accuracy of transcriptome abundance calculations [29,30]. Overcoming these drawbacks, third-generation PacBio sequencing technology yields full-length reads with uniform coverage, offering significant advantages in constructing comprehensive transcriptomes. Here, we integrate data from full-length transcriptome sequencing and RNA-seq of Hyb and its parental species for the first time, shedding light on molecular-level transcriptomic changes underlying growth heterosis in Hyb.

## Materials and methods

### Sample preparation and ethics statement

Three-year-old Cal, Ela and Hyb were used as experimental samples. They were cultivated under the same breeding conditions at Hainan Chenhai Aquatic Co., Ltd. in Hainan Province, China. Five individuals of each species were selected randomly. The mean body weight was 0.46±0.03 kg, 2.68±0.07 kg and 1.84±0.4 kg in Cal, Ela and Hyb, respectively. The brain, liver, and muscle tissues were dissected from each fish after euthanasia by immersion in MS-222 buffered solution (3 g/L) on ice. All experimental procedures strictly adhered to the guidelines stipulated by Administration of Affairs Concerning Animal Experimentation of China and were approved by the Institutional Review Board on Bioethics and Biosafety of BGI (FT14015).

### Full-length transcriptome sequencing

Total RNA was extracted from the three tissues (brain, liver, and muscle) using a TRIzol kit (Invitrogen) according to the manufacturer’s instructions. Total RNA quality was determined using an Agilent 2100 Bioanalyzer, and RNA integrity was analyzed for subsequent experiments. The concentration of RNA in each sample was greater than 300 ng/ul, and the total amount of the RNA used for the cDNA library was greater than 5 ug. NEBNext®Single Cell/Low Input cDNA Synthesis & Amplification Module was used to synthesize full-length cDNA. The harvested cDNA was processed for end-repair/A tailing. Finally, single-molecule real-time (SMRT) hairpin adapters were ligated to the ends of double-stranded cDNA molecules. The purity, concentration, and insert size of the library were assessed to ensure library quality; qualified libraries were processed for full-length transcriptome sequencing on the PacBio sequencing platform. Full-length transcripts were obtained in three main steps: full-length sequence identification, consensus sequence clustering, and consensus sequence polishing [31]. Corrected transcript sequences were clustered using CD-HIT 4.6.7 software based on 95% similarity between the sequences [32]. The benchmarking universal single-copy ortholog (BUSCO) 3.0.2 software was employed to evaluate the completeness of the full-length transcriptome sequence [33].

### Next-generation sequencing

The amount of 1 μg RNA per sample was used for RNA-seq library construction. First-strand cDNA was synthesized using fragmented mRNA as a template and random hexamers as primers, followed by second-strand synthesis with the addition of RNase H and DNA polymerase I. cDNA was purified using AMPure XP beads. Double-stranded cDNA was then subjected to end repair. Adenosine was added to the end and ligated to the adapters. AMPure XP beads were used to select fragments within a specific size range. Transcriptome sequencing was performed using the Illumina NovaSeq6000 sequencing platform. The full-length non-chimeric transcript per species was used as a reference sequence to obtain the isoform after removing redundancy with CD-HIT, and STAR software was used to compare the next-generation sequencing data with the above reference sequence. Gene expression levels were quantified using Kallisto, based on transcripts generated from circular consensus data [34]. Fragments Per Kilobase of transcript per million mapped reads (FPKM) were used as the standard method to estimate gene expression levels. Raw data have been submitted to GenBank database (BioProject ID: PRJNA1112737, https://dataview.ncbi.nlm.nih.gov/object/PRJNA1112737?reviewer=qa7p8lskotat189o94h7oeuue1).

### Differential expression and cluster analysis

Differential expression analysis was performed using edgeR (version 4.0.16) based on read counts. The screening criteria of differentially expressed genes (DEGs): FDR < 0.01 and | log2(Fold Change)| ≥ 2.5 were set as the threshold. Analysis of similarities (ANOSIM) and principal coordinate analysis (PCoA) were implemented for DEGs using the vegan (2.6–4) and ape packages (5.7–1) [35]. Clustering analyses were conducted to search for DEGs with similar expression behavior using the upset R package 1.4.0 and the pheatmap v1.0.12 package. The ggplot2 R package was used to draw pictures [36].

### Function and pathway analysis

All DEGs in different tissues were submitted to DAVID (https://david.ncifcrf.gov/summary.jsp) for enrichment analysis of the differential expression gene by Gene Ontology (GO) number, GO biological processes and molecular function terminology. The relationships among the DEGs were predicted using correlation network analysis. Corr. was used to calculate the correlation coefficient using the psych package (version 1.9.6). When the correlation coefficient (r) was > 0.2, *P* < 0.001 was considered significant and listed. *P*-value was inferred by adjusting the *p*-value using the Benjamini and Hochberg’s method [37]. The igraph v2.0.2 was used for network analysis, and Gephi v0.10.1 realized the network visualization.

Based on publicly available pathway databases, the Kyoto Encyclopedia of Genes and Genomes (KEGG) pathway mapper (https://www.kegg.jp/kegg/mapper/) was used to identify the metabolic pathways of genes related to growth traits.

### Data analysis

To verify the results of the transcriptional sequencing, the real-time quantitative PCR (qRT-PCR) was conducted on the brain, liver, and muscle tissues of Hyb and its parents (primers were listed in S1 Table). The 2^−ΔΔCt^ method [38] was applied to determine the gene expression abundance. All other data were analyzed using SPSS17.0 software.

## Results

### Data analysis of full-length transcriptome sequencing and next-generation sequencing

**Fig 1:**
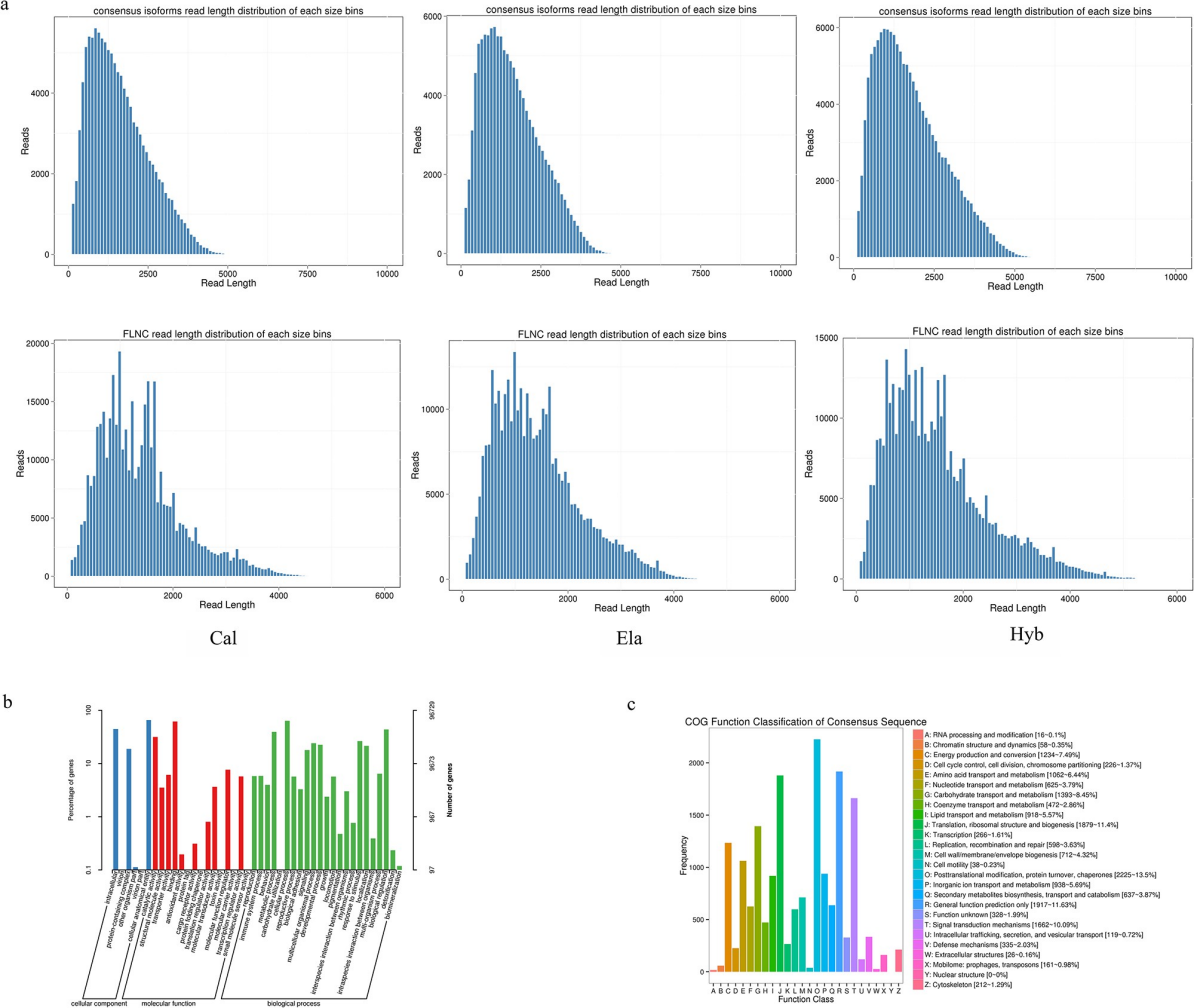
Full-length transcriptome sequencing, assembly, and annotation in Cal, Ela and Hyb. (a) Length distribution of the consensus isoform and full-length non-chimeric read (FLNC). (b) Gene Ontology (GO) classification of the assembled full-length transcripts. (c) Cluster of Orthologous Groups of proteins (COG) classification of the assembled full-length transcripts.

Full-length transcriptome data for Cal, Ela, and Hyb were obtained using the PacBio Sequel platform, with 44.70Gb, 40.44Gb, and 45.32Gb of SMRT sequencing data, which generated 481,134, 440,996, and 494,591 circular consensus (CCS) reads, respectively. After filtering out incomplete circular consensus reads, 399,162, 337,744, and 392,975 full-length non-chimeric (FLNC) sequences with complete 5’-3’ ends were obtained, which correspond to Cal, Ela, and Hyb. After removing redundant sequences, 74,516, 77,419, and 98,523 consensus sequences were obtained in Cal, Ela, and Hyb by CD-HIT, respectively, and then combined into 204,322 non-redundant isoforms (Fig 1A). Based on BUSCO analysis, 70.29% (Cal), 71.95% (Ela), and 75.58% (Hyb) of the complete orthologs were identified (S2 Table). A total of 78,672 complete coding sequences (CDS) were predicted (Table 1).

**Table 1:** Summary for the transcriptome data of Hyb and its parents using PacBio.

	Cal			Ela	Hyb
SMRT sequencing data (Gb)	44.70			40.44	45.32
Number of CCS	481,134			440,996	494,591
Read Bases of CCS	789,356,562			737,852,331	885,804,403
Mean Read Length of CCS	1,640			1,673	1,790
Number of undesired primer reads	70,482			90,483	85,056
FLNC	399,162			337,744	392,975
FLNC%	82.96%			76.59%	79.45%
Number of consensus sequences	119,760			124,978	145,361
Average consensus isoforms read length		1,589	1,584		1,765
Number of consensus sequences with removing redundant sequences		74,516	77,419		98,523
Number of combined non-redundant isoforms	204,322				
Number of complete CDS	78,672				

In this study, 125,305 reads were annotated as combined non-redundant FLNC reads based on seven databases. The number of annotated reads in the seven databases ranged from 93,622 (45.82%; KEGG pathways) to 120,463 (58.96%; Non-Redundant Protein (NR) database) (S3 Table). In addition, 96,729 reads were annotated in the GO database (Fig 1B), and 16,541 reads were annotated in the Clusters of Orthologous Groups of proteins (COG) database (Fig 1C).

A total of 953,794,305 clean reads were generated using the Illumina platform with an average of 21,195,429 reads per library. All clean reads were mapped to full-length transcriptome databases of Cal, Ela, and Hyb using Spliced Transcripts Alignment to a Reference (STAR). The final alignment comprised 838,451,691 reads, and the average mapping rate of all RNA-seq libraries was 87.91%, indicating that the PacBio library had a high degree of integrity (S4 Table).

### Identification DEGs and enrichment analyses

**Fig 2:**
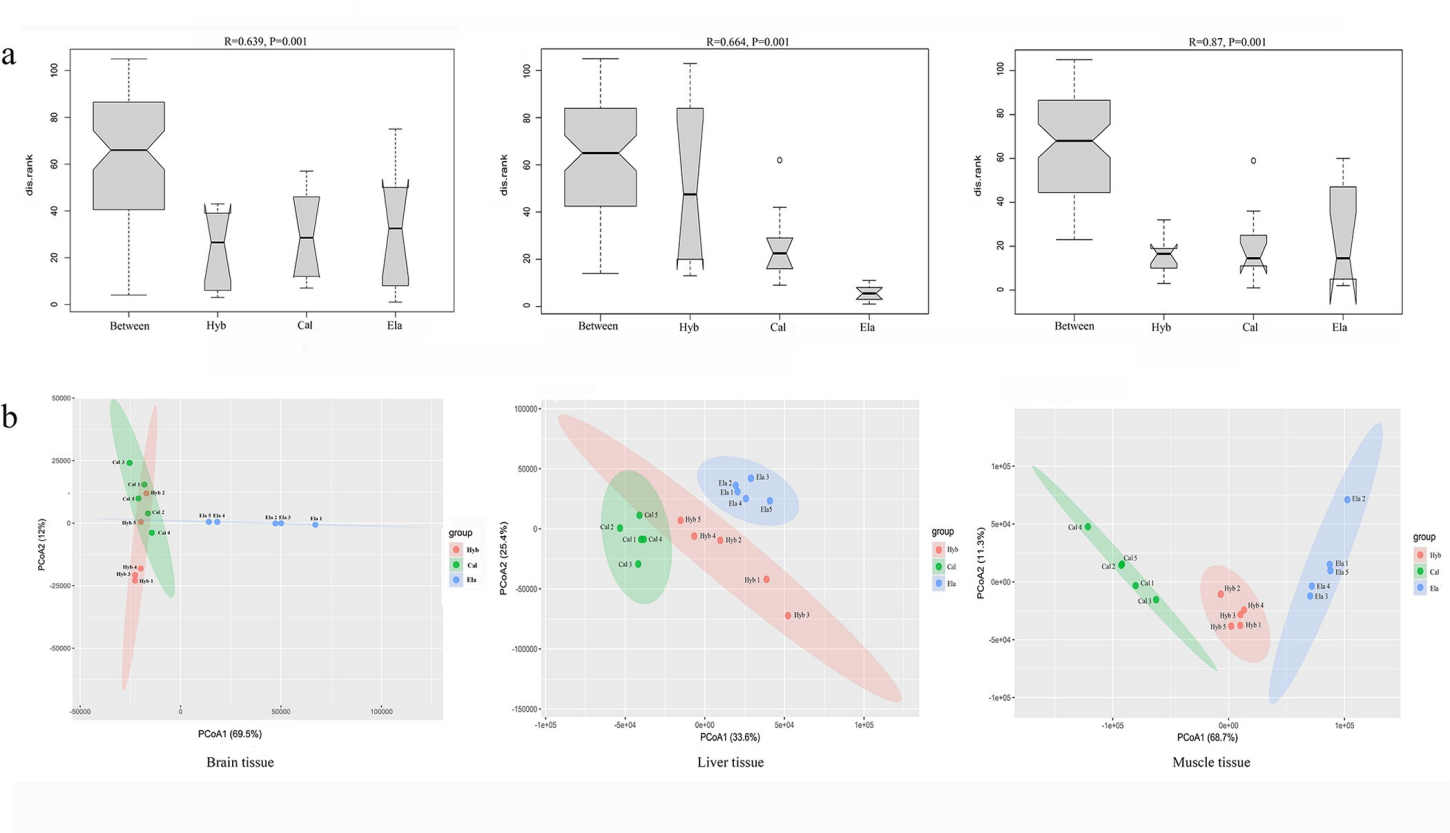
Analysis of similarities (ANOSIM) and principal coordinates analysis (PCoA) of Cal, Ela and Hyb in brain tissue (a), liver tissue (b) and muscle tissue (c), respectively.

ANOSIM showed that the difference between species was greater than that within species (R = 0.639, *p* = 0.001 in brain tissue; R = 0.664, *p* = 0.001 in liver tissue; and R = 0.87, *p* = 0.001 in muscle tissue), indicating that statistical significance existed in the comparison between different species (Fig 2A). PCoA showed that different individuals of each species could be clearly clustered or grouped, indicating good repeatability within the species. The differences in transcription levels in the muscle tissue were the greatest among the three species (Fig 2B).

**Fig 3:**
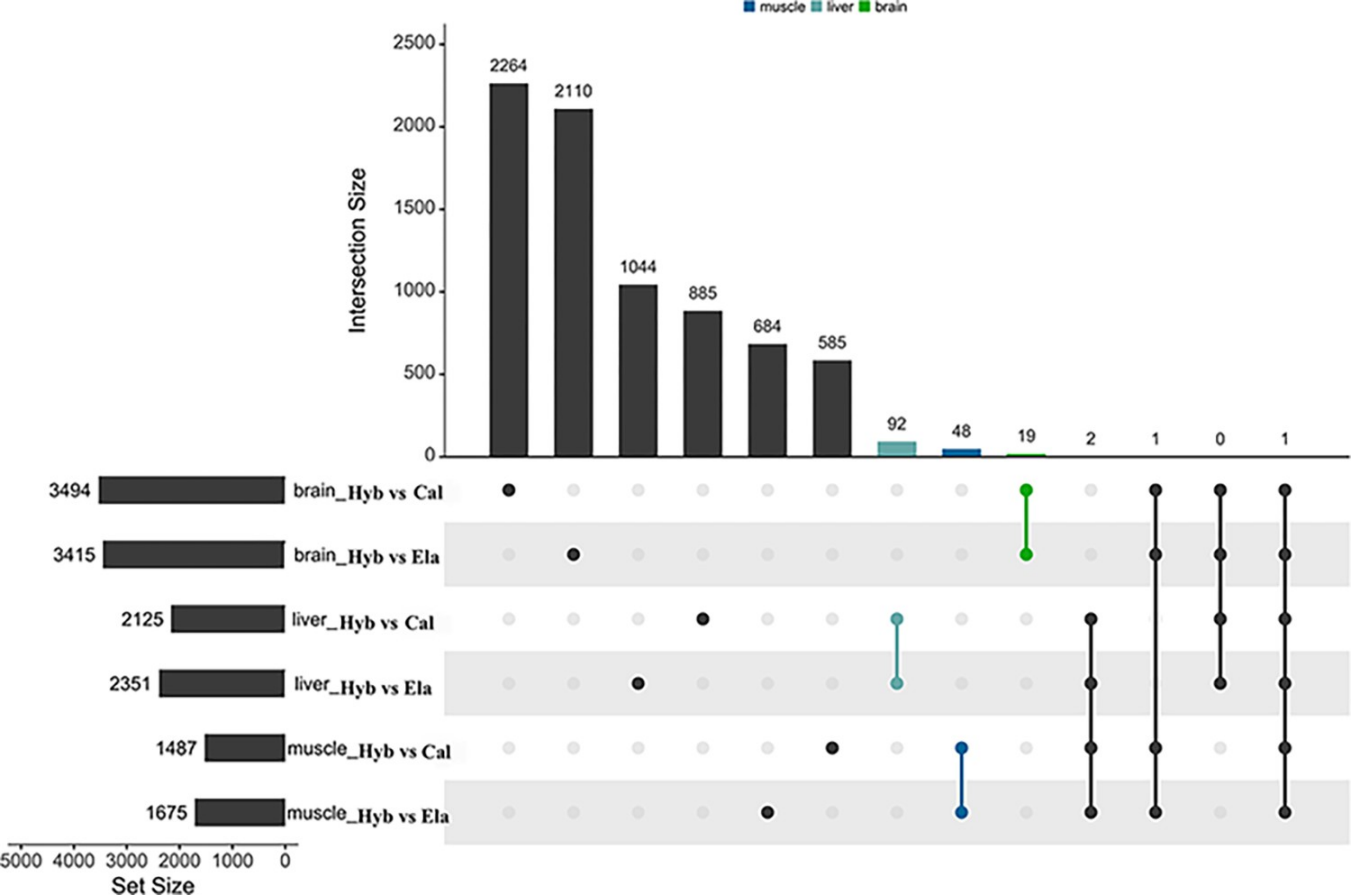
UpSet plot representation of intersection regions of the different comparison groups in brain, liver and muscle tissue.

**Fig 4:**
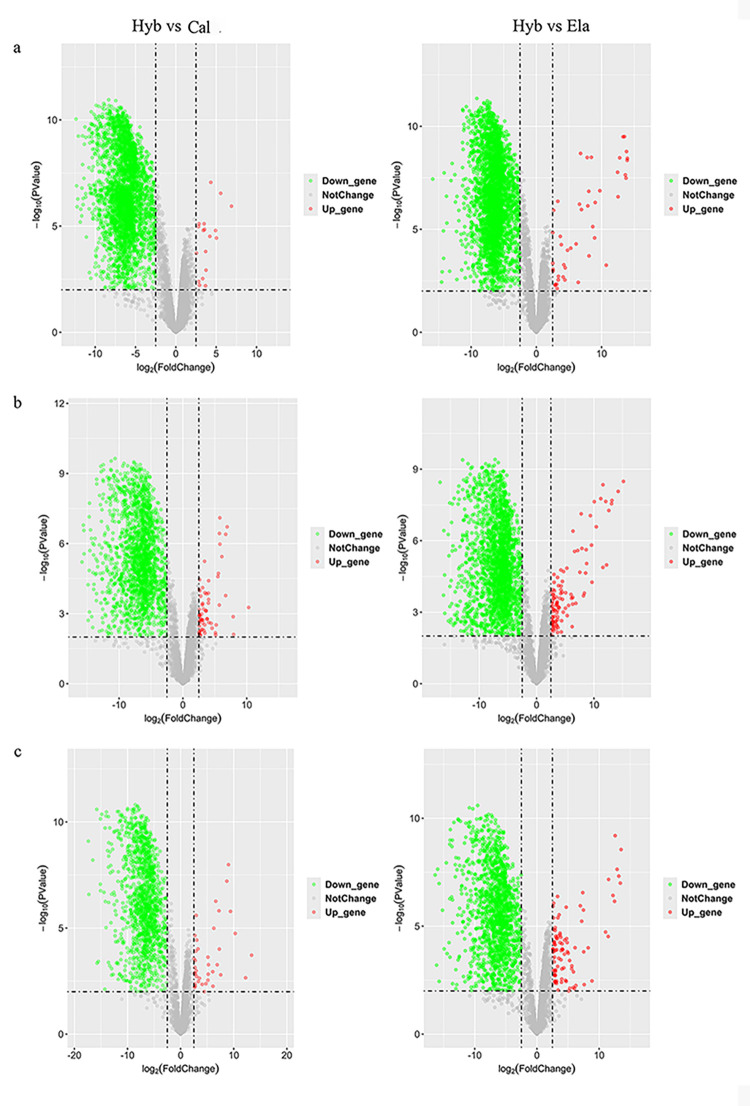
Volcano plot showing differentially regulated genes between Hyb vs Cal, Hyb vs Ela in brain tissue (a), liver tissue (b) and muscle tissue (c), respectively.

To fully compare the differences between the hybrid groupers and parents, we set up two comparisons: Hyb vs. Cal and Hyb vs. Ela. In the brain, there were 3,494 differences between Hyb and Cal (18 upregulated, 3,476 downregulated) and 3,415 differences between Hyb and Ela (45 upregulated, 3,370 downregulated) (Figs 3 and 4A). The number of common DEGs that only exist between the two comparisons was 19. In the liver tissue, 2,125 (66 upregulated, 2,059 downregulated) and 2,351 (106 upregulated, 2,245 downregulated) DEGs were found in the Hyb vs. Cal and Hyb vs. Ela comparisons, respectively (Figs 3 and 4B). Only 92 common DEGs were found between these two comparisons in liver tissue. In muscle tissue, 1,487 (38 upregulated, 1,449 downregulated) and 1,675 (93 upregulated, 1,582 downregulated) DEGs were identified in Hyb vs. Cal and Hyb vs. Ela comparisons, respectively (Figs 3 and 4C). Only 48 common DEGs were detected between the two comparisons in the muscle tissue.

**Fig 5:**
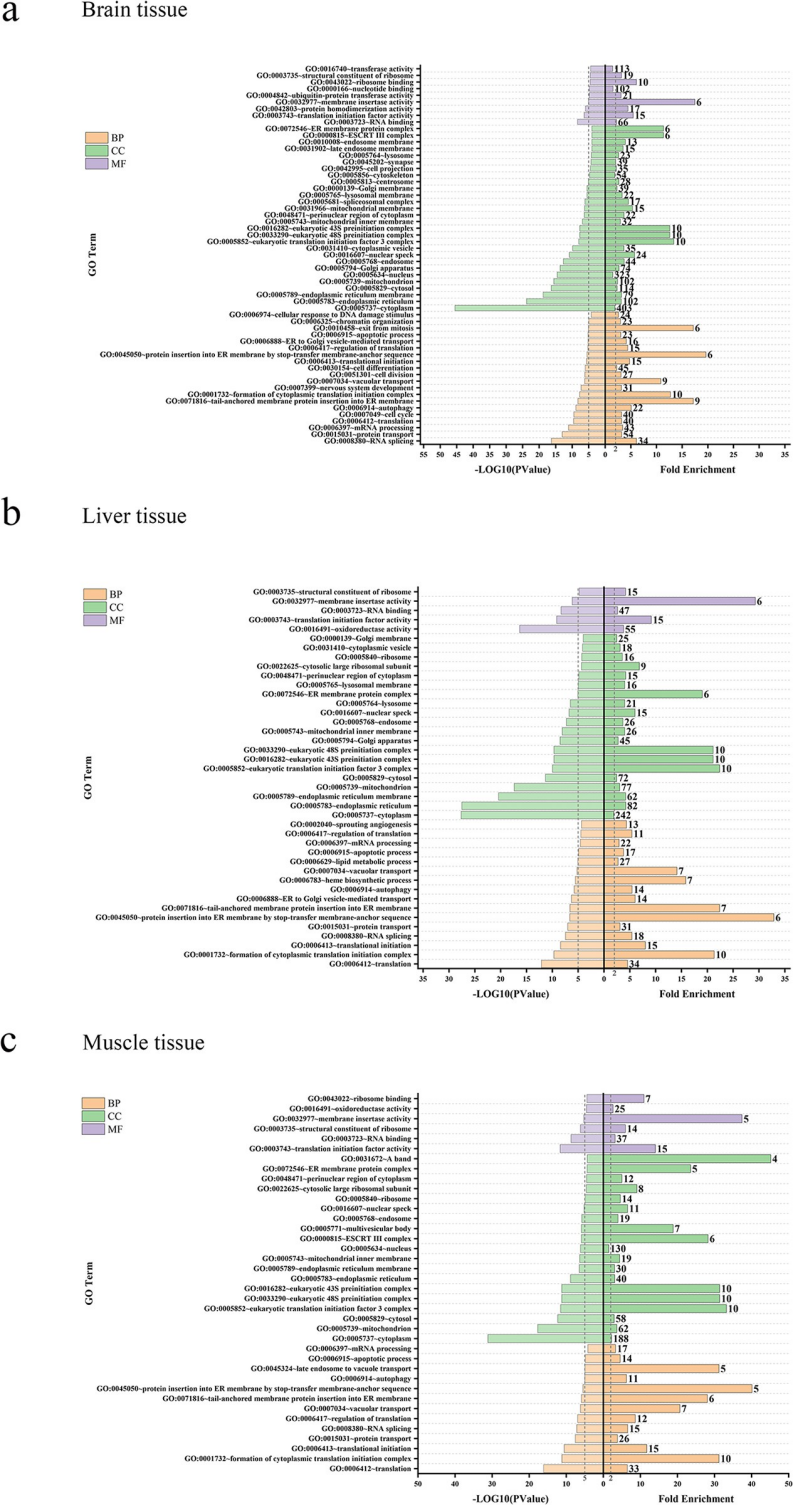
GO enrichment of differentially expressed genes (DEGs) in brain tissue (a), liver tissue (b) and muscle tissue (c), respectively.

The GO analysis of the total DEGs produced three major functional categories: cellular component (CC), molecular function (MF), and biological processes (BP). In CC, the cytoskeleton was significantly enriched in brain tissue. For BP, most of the enriched subcategories were related to protein transport in the brain, liver, and muscle tissues (Fig 5).

### Clustering and correlation network analysis of DEGs

**Fig 6:**
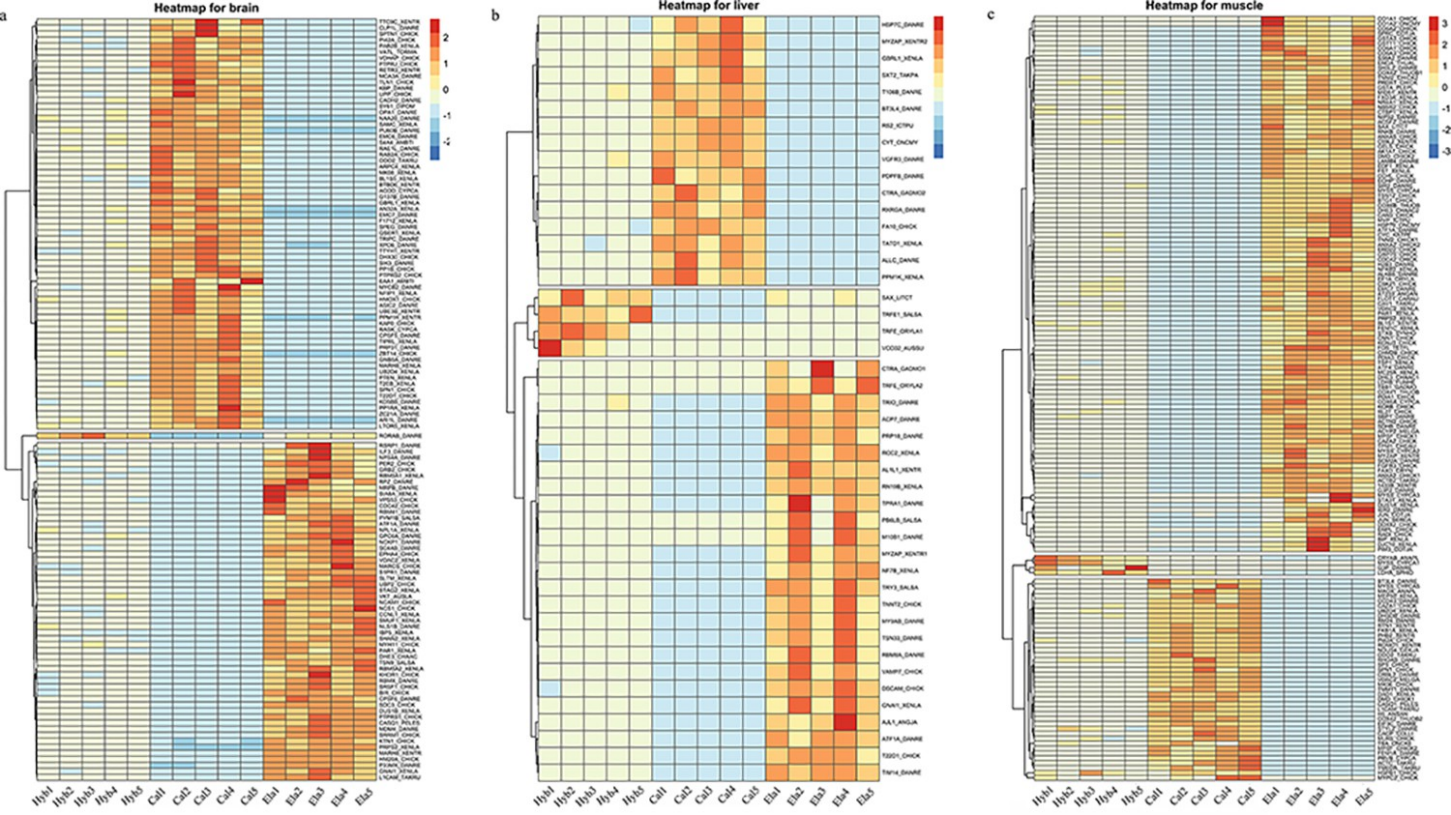
Heatmap of DEGs in brain tissue (a), liver tissue (b) and muscle tissue (c), respectively.

According to the relative expression trends, the DEGs were mainly categorized into three groups: those with high expression in Cal, high expression in Ela, and high expression in Hyb (Fig 6). In the liver tissue, the number of DEGs was the lowest (Fig 6B). In all tissues, more DEGs were highly expressed in Cal or Ela, while the expression levels of DEGs in the Hyb were intermediate.

**Fig 7:**
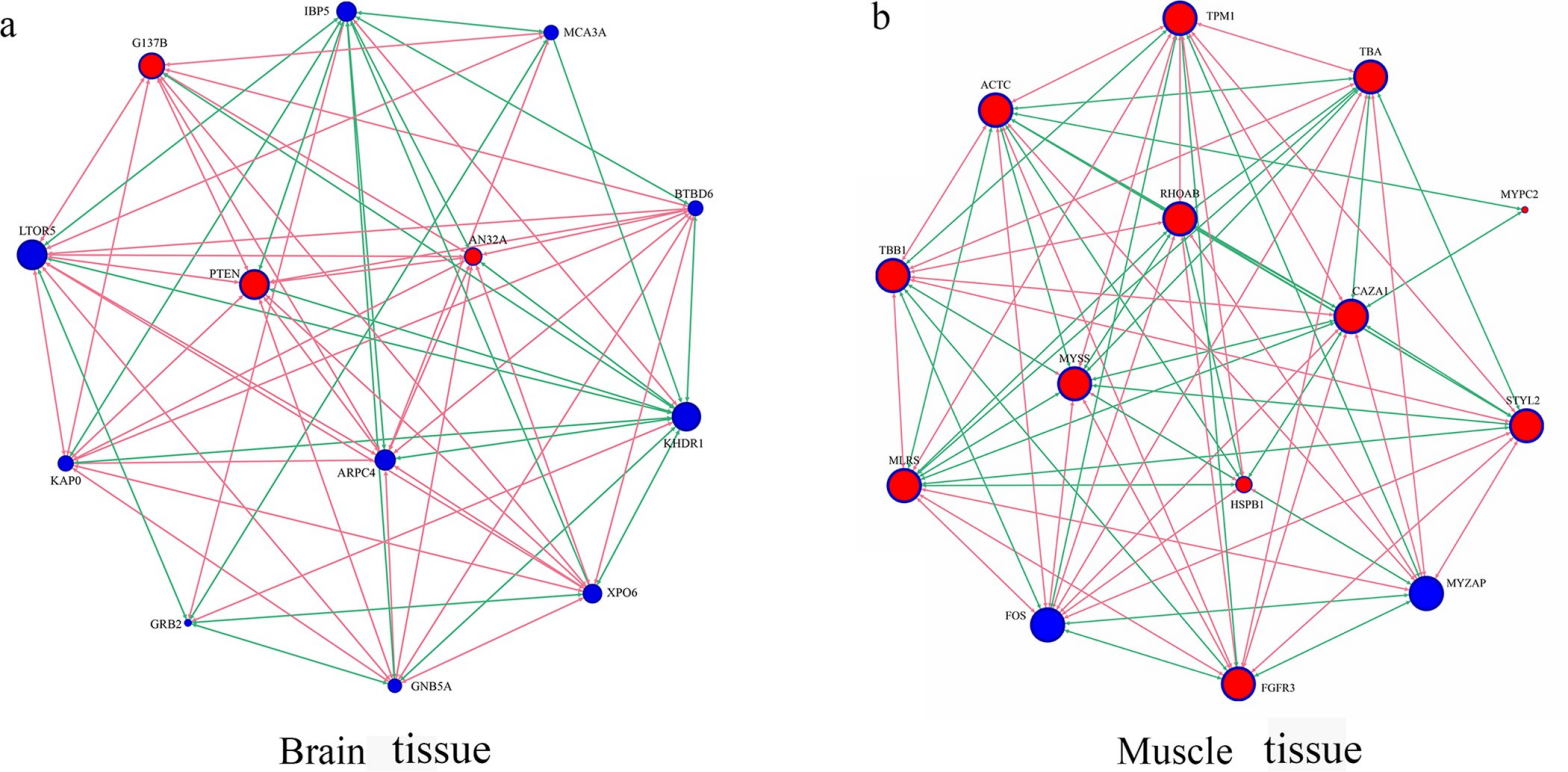
Construction of the growth-related genes relationship network through correlation network analysis in brain tissue (a) and muscle tissue (b). Red arrow indicates positive regulation, green arrow indicates negative regulation. Red node shows the expression level trend of this gene is consistent with transcriptome sequencing (RNA-seq). Blue node shows the expression level trend of this gene is not consistent with RNA-seq.

**Fig 8:**
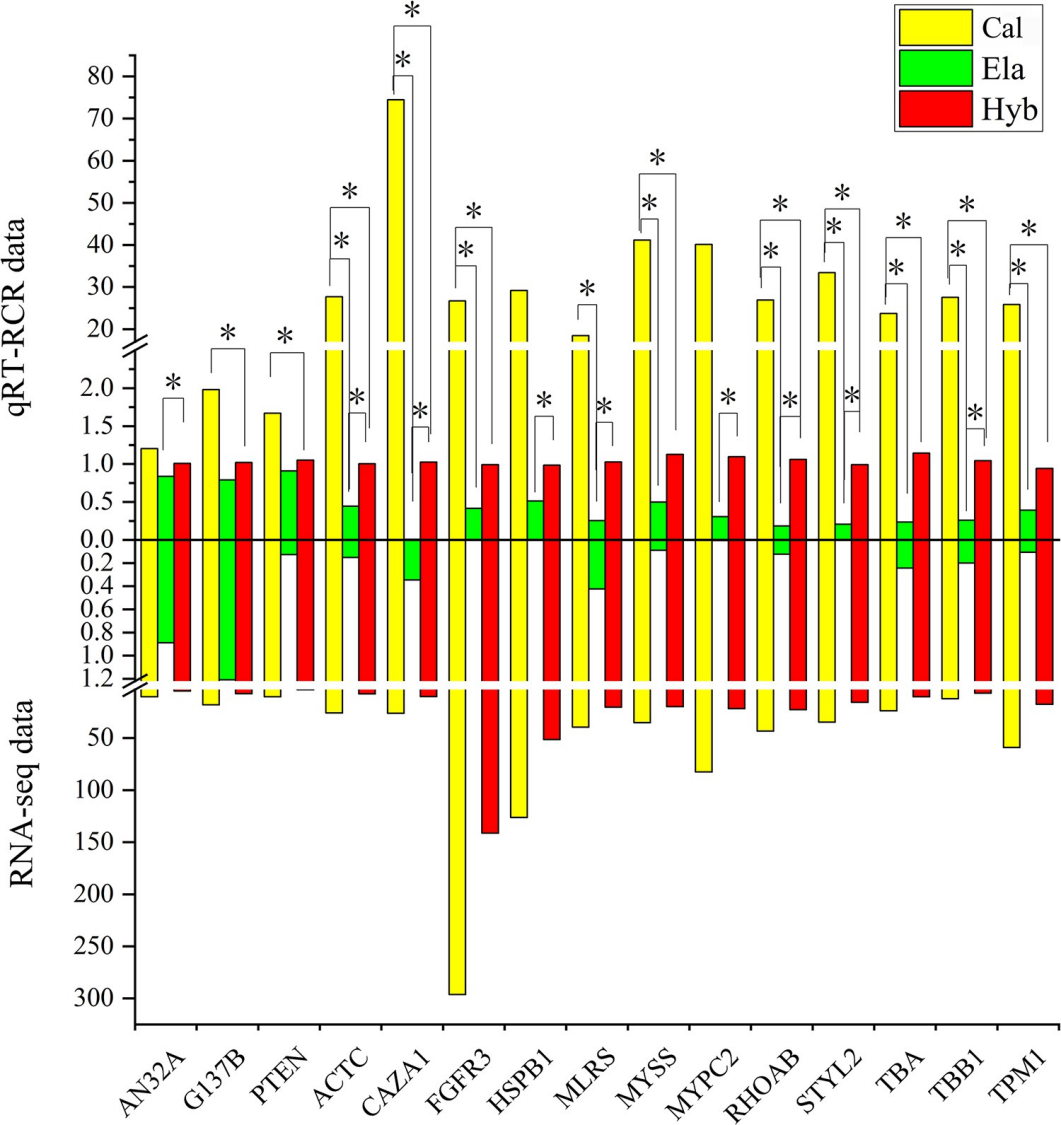
Real-time quantitative PCR (qRT-PCR) validation of candidate genes. Asterisk represents there is a significant difference between the two samples, as assessed by a t-test (p < 0.05).

The correlation analysis to screen genes related to growth traits and explore their regulatory relationships found that 13 growth-related DEGs were detected in the brain tissues and 14 growth-related DEGs were detected in the muscle tissues. These DEGs formed a network in the brain and muscle tissues, respectively (Fig 7). There were two growth-related DEGs (MYZAP and TNNT2) found in liver tissue, but they did not form a network. The qRT-PCR expression patterns of the 15 DEGs were consistent with the data obtained from RNA-seq (AN32A, G137B, and PTEN from brain tissue; ACTC, CAZA1, FGFR3, HSPB1, MLRS, MYSS, MYPC2, RHOAB, STYL2, TBA, TBB1, and TPM1 from muscle tissue) (Fig 8 and S5 Table). The relative expression levels of the 15 DEGs in Hyb were lower than those in Cal and higher than those in Ela. Unfortunately, no DEGs with similar expression patterns between qRT-PCR and RNA-seq were found in the liver tissue.

### Pathway analysis

**Fig 9:**
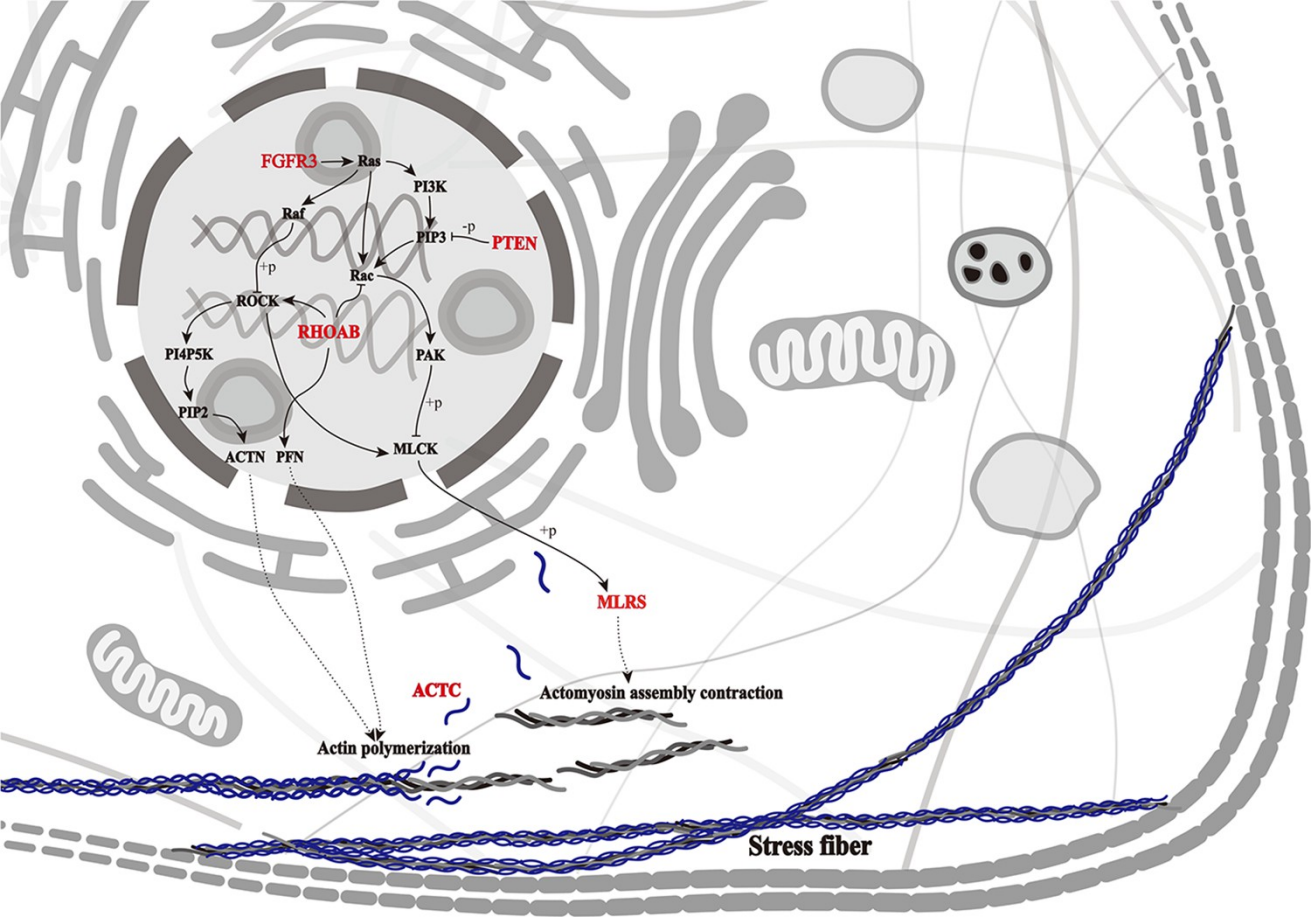
The predicted growth signaling pathway in Hyb based on the regulation of actin cytoskeleton of the Kyoto Encyclopedia of Genes and Genome (KEGG) pathway database. Genes marked red are verified by qRT-PCR.

Two genes (PTEN and HSPB1) were enriched in the MAPK signaling pathway. Five genes (PTEN, FGFR3, RHOAB, MLRS, and ACTC) were involved in the regulation of the actin cytoskeleton, forming the primary enrichment pathway (Fig 9). In addition, only PTEN was differentially expressed in the brain tissue, whereas other genes were differentially expressed in the muscle tissue. These findings suggest that PTEN may be a key upstream regulatory gene in Hyb growth.

## Discussion

Growth is a complex trait controlled by multiple genes within multiple organs. The complex genetic mechanisms associated with growth in groupers have attracted the attention of many researchers. RNA-seq technology can help acquire further insight into the molecular regulation mechanisms of biological processes by analyzing a variety of total transcript categories, structures, and expression levels [39,40]. RNA-seq is widely used to identify regulatory factors related to growth traits in groupers [41–43]. In this study, we adopted a combined strategy of RNA-Seq and SMRT sequencing to analyze the mechanism of Hyb growth superiority to correct the shortcomings of using RNA-seq alone.

Comparative transcript analysis between Hyb and its parents indicated that DEGs were related to growth traits, primarily in muscle and brain tissues. No DEGs were found in the liver tissue based on qRT-PCR results. These observations indicate that the brain and muscle are the main tissues controlling the growth of Hyb. Interestingly, the expression pattern of DEGs validated by qRT-PCR in Hyb was consistent, with the relative expression levels being higher than those in Ela and lower than those in Cal. In yeast, it was found that most genes whose expression differed between the hybrid and its parents displayed intermediate expression levels between the two parents [44]. In *Arabidopsis*, gene expression in the hybrids also exhibited intermediate levels between the two parents or was close to the level of one of the two parents. It has been proposed that gene expression levels are subjected to natural selection because living organisms must balance the costs and benefits of protein production and activity [45,46]. Intermediate expression levels in hybrids may further result in better resource efficiency, resulting in heterosis [44,47]. The findings from 16 maize hybrids, according to genome-wide expression analysis, imply a positive association between the proportion of genes showing mid-parent expression and yield heterosis [48].

KEGG pathway enrichment analysis revealed that these growth-related DEGs were enriched in the MAPK pathway and actin cytoskeleton regulation. MAKPs are kinase families that regulate myogenic cell proliferation, differentiation, and protein synthesis in fish [5]. The MAPK/ERK signaling pathway can promote muscle cell proliferation [49] and terminal differentiation [50], which can be activated by IGFs. Actin polymerization and cytoskeletal remodeling are closely associated with smooth muscle contraction. Dynamic changes in the actin cytoskeleton play a fundamental role in the regulation of tension development during smooth muscle contraction [18]. The p70^S6k^, as a potent stimulator of protein synthesis, is activated by increasing muscle contraction [16]. Activated p70^S6k^ can promote muscle growth via the IGF-I/Akt/mTOR pathway [51]. Therefore, it can be inferred that Hyb growth is regulated by the GH/IGF system. The contribution of the GH/IGF system and its downstream signaling pathways in regulating growth superiority has been reported in other fish [5,42]. We also found that PTEN was enriched in two pathways, suggesting that it plays a vital role in the regulation of Hyb growth. PTEN is a crucial gene in regulating cell growth and division and executes its biological function depending on the Akt/PKB and PI3K/Akt signaling pathways [52,53]. PTEN has been generally studied in humans but rarely in fish. Our results indicate that PTEN plays an important role in the regulation of growth through the MAPK pathway and actin cytoskeleton. However, the specific mechanism of PTEN action requires further investigation.

## Supporting information

S1 Table The primers used for qRT-PCR. (DOC)

S2 Table The results of the transcriptome integrity assessment based on BUSCO using aneukaryota (ODB9) core gene dataset. (DOC)

S3 Table BLAST analysis of the full-length transcripts against public databases. (DOC)

S4 Table Summary of the alignment between next-generation sequencing reads and PacBio transcripts. (DOC)

S5 Table Gene annotation of DEGs in the growth-related correlation network. (DOC)

## References

[1] Tran Trang Thi Huyen, Nguyen Hoa Thi, Le Binh Thi Nguyen, Tran Phuc Huu, Nguyen Sang Van, Kim Oanh Thi Phuong. Characterization of single nucleotide polymorphism in IGF1 and IGF1R genes associated with growth traits in striped catfish (Pangasianodon hypophthalmus Sauvage, 1878). Aquaculture. 2021; 538.

[2] Zhu F, Sun H, Jiang L, Zhang Q, Liu J. Genome-wide association study for growth-related traits in golden pompano (Trachinotus ovatus). Aquaculture. 2023; 739549.

[3] Zhang C, Wen H, Zhang Y, Zhang K, Qi X, Li Y. First genome-wide association study and genomic prediction for growth traits in spotted sea bass (Lateolabrax maculatus) using whole-genome resequencing. Aquaculture. 2023; 566:739194.

[4] Yu Hui, Xinxin You, Li J Zhang XH, Zhang S Jiang SJ, et al. A genome-wide association study on growth traits in orangespotted grouper(Epinephelus coioides) with RAD-seq genotyping. Science China (Life Sciences). 2018; 61:934–946. doi: 10.1007/s11427-017-9161-4 29541990

[5] Fuentes EN, Valdés JA, Molina A, Björnsson BT. Regulation of skeletal muscle growth in fish by the growth hormone-Insulin-like growth factor system. General and Comparative Endocrinology. 2013; 192: 136–148. doi: 10.1016/j.ygcen.2013.06.009 23791761

[6] Björnsson BT, Johansson V, Benedet S, Einarsdottir IE, Hildahl J, Agustsson T, et al. Growth Hormone Endocrinology of Salmonids: Regulatory Mechanisms and Mode of Action. Fish Physiology & Biochemistry. 2002; 27:227–242.

[7] LeRoith D, Bondy C, Yakar S, Liu JL, Butler A. The somatomedin hypothesis. Endocr. Rev. 2001; 22: 53–74. 11159816 10.1210/edrv.22.1.0419

[8] Duan C, Ding J, Li Q, Tsai W, Pozios K. Insulin-like growth factor binding protein 2 is a growth inhibitory protein conserved in zebrafish. Proc. Natl. Acad. Sci. USA. 2010; 96:15274–15279. 10.1073/pnas.96.26.15274 PMC24810 10611375

[9] Wood AW, Duan C, Bern HA. Insulin-Like Growth Factor Signaling in Fish. Int. Rev. Cytol. 2005; 243:215–285. doi: 10.1016/S0074-7696(05)43004-1 15797461

[10] Hagemeister AL, Sheridan MA. Somatostatin inhibits hepatic growth hormone receptor and insulin-like growth factor I mRNA expression by activating the ERK and PI3K signaling pathways. Am J Physiol Regul Integr Comp Physiol. 2008; 295:490–497. doi: 10.1152/ajpregu.00099.2008 18495839

[11] Sheridan MA, Hagemeister AL. Somatostatin and somatostatin receptors in fish growth. Gen Comp Endocrinol. 2010; 167:360–365. doi: 10.1016/j.ygcen.2009.09.002 19735661

[12] Sun Y, Huang Y, Hu G, Zhang X, Ruan Z, Zhao X, et al. Comparative Transcriptomic Study of Muscle Provides New Insights into the Growth Superiority of a Novel Grouper Hybrid. Plos One. 2016; 11: e0168802. 10.1371/journal.pone.0168802 PMC5179234 28005961

[13] Pierce AL, Fox BK, Davis LK, Visitacion N, Kitahashi T, Hirano T, et al. Prolactin receptor, growth hormone receptor, and putative somatolactin receptor in Mozambique tilapia: tissue specific expression and differential regulation by salinity and fasting. General & Comparative Endocrinology. 2007; 154: 31–40. doi: 10.1016/j.ygcen.2007.06.023 17714712

[14] Reindl KM, Sheridan MA. Peripheral regulation of the growth hormone-insulin-like growth factor system in fish and other vertebrates. Comparative Biochemistry & Physiology Part A. 2012; 163:231–245. doi: 10.1016/j.cbpa.2012.08.003 22909791

[15] Weatherley AH, Gill HS. Dynamics of increase in muscle fibers in fishes in relation to size and growth. Experientia. 1985; 41:353–354.

[16] Barr K, Esser K. Phosphorylation of p70S6k correlates with increased skeletal muscle mass following resistance. American Journal of Physiology Cell Physiology. 1999; 276:120–127. 10.1152/ajpcell.1999.276.1.C120 9886927

[17] Tidball JG. Mechanical signal transduction in skeletal muscle growth and adaptation. J Appl Physiol. 2005; 98:1900–1908. doi: 10.1152/japplphysiol.01178.2004 15829723

[18] Gunst SJ, Zhang W. Actin cytoskeletal dynamics in smooth muscle: A new paradigm for the regulation of smooth muscle contraction. AJP Cell Physiology. 2008; 295:C576–C587. doi: 10.1152/ajpcell.00253.2008 18596210 PMC2544441

[19] FAO. FAO yearbook. In: Fishery and Aquaculture Statistics 2022/FAO Annuaire. FAO, Rome. 2022.

[20] Wang Q, Liu Y, Peng C, Wang X, Xiao L, Wang D, et al. Molecular regulation of sex change induced by methyltestosterone -feeding and methyltestosterone -feeding withdrawal in the protogynous orange-spotted grouper,. Biology of Reproduction. 2017; 97:324–333. doi: 10.1093/biolre/iox085 29044430

[21] James CM, Al-Thobaiti SA, Rasem BM, Carlos MH. Potential of grouper hybrid (Epinephelus fuscoguttatus x E. polyphekadion) for aquaculture. ICLARM International Center for Living Aquatic Resources Management Quarterly. 1999; 22:19–23.

[22] Chen S, Tian YS, Li ZQ, Li ZT, Zhang JJ, Wang LN, et al. Metamorphosis and skeletal development of hybrid Epinephelus awoara (♀) and Epinephelus tukula (♂) progenies. Aquaculture. 2021; 530: 735727–735737.

[23] Kubota S, Longloy A, Singhabun A, Khammee W, Kessuwan K, Bunlipatanon P, et al. Quantitative trait locus mapping of growth-related traits in inter-specific F 1 hybrid grouper (Epinephelus fuscoguttatus × E. lanceolatus) in a tropical climate. Aquaculture Research. 2017; 48.

[24] Chen J, Ye Z, Yu Z, Wang J, Li P, Chen X, et al. The complete mitochondrial genome of the hybrid grouper (Cromileptes altivelis♀ × Epinephelus lanceolatus♂) with phylogenetic consideration. Mitochondrial Dna Part B. 2017; 171–172. doi: 10.1080/23802359.2017.1303346 33473756 PMC7799962

[25] Cao L, Chen P, Hou X, Ma J, Yang N, Lu Y, et al. rDNA and mtDNA analysis for the identifcation of genetic characters in the hybrid grouper derived from hybridization BMC Genom Data. 2024; 25. 10.1186/s12863-023-01188-5 PMC10787421 38216865

[26] Luo H, Ye H, Xiao S, Zheng S, Wang X, Wang Z. Application of transcriptomics technology to aquatic animal research. Journal of Fisheries of China. 2015; 39: 598–607.

[27] Zhou K, Zhang K, Fan X, Zhang W, Liang Y, Wen X, et al. The skin-color is associated with its physiological state: A case study on a colorful variety, hybrid grouper (Epinephelus fuscoguttatus × Epinephelus lanceolatus). Aquaculture. 2022; 549:737719.

[28] Zhu X, Hao R, Tian C, Zhang J, Zhu C, Li G. Integrative Transcriptomics and Metabolomics Analysis of Body Color Formation in the Leopard Coral Grouper (Plectropomus leopardus). Frontiers in Marine Science. 2021; 8:726102.

[29] Liang Y, Chen S, Liu G. Application of next generation sequencing techniques in plant transcriptome. Yi Chuan. 2011; 33:1317–1326. doi: 10.3724/sp.j.1005.2011.01317 22207377

[30] Zhu Z, Chen H, Xie K, Liu C, Li L, Liu L, et al. Characterization of Drought-Responsive Transcriptome During Seed Germination in Adzuki Bean (Vigna angularis L.) by PacBio SMRT and Illumina Sequencing. Frontiers in Genetics. 2020; 11:996. doi: 10.3389/fgene.2020.00996 33110419 PMC7489039

[31] Li W, Adam G. Cd-hit: a fast program for clustering and comparing large sets of protein or nucleotide sequences. Bioinformatics. 2006; 22:1658–1659. doi: 10.1093/bioinformatics/btl158 16731699

[32] Fu L, Niu B, Zhu Z, Wu S, Li W. CD-HIT: accelerated for clustering the next-generation sequencing data. Bioinformatics. 2012; 28:3150–3152. doi: 10.1093/bioinformatics/bts565 23060610 PMC3516142

[33] Seppey M, Manni M, Zdobnov EM. BUSCO: Assessing Genome Assembly and Annotation Completeness. Methods Mol Biol. 2019; 1962:227–245. doi: 10.1007/978-1-4939-9173-0_14 31020564

[34] Bray NL, Pimentel H, Melsted P, Pachter L. Near-optimal probabilistic RNA-seq quantification. Nat Biotechnol. 2016; 34:525–527. doi: 10.1038/nbt.3519 27043002

[35] Price AL, Patterson NJ, Plenge RM, Weinblatt ME, Shadick NA, Reich D. Principal components analysis corrects for stratification in genome-wide association studies. Nature Genetics. 2006; 38:904–909. doi: 10.1038/ng1847 16862161

[36] Ginestet C. ggplot2: Elegant Graphics for Data Analysis. Journal of the Royal Statistical Society Series A: Statistics in Society. 2011; 174:245–246.

[37] Benjamini Y, Hochberg Y. Controlling the False Discovery Rate: A Practical and Powerful Approach to Multiple Testing. Journal of the Royal Statistical Society. Series B: Methodological. 1995; 57:289–300.

[38] Livak KJ, Schmittgen TD. Analysis of relative gene expression data using real-time quantitative PCR and the 2(-Delta Delta C(T)) Method. Methods. 2001; 25:402–408. doi: 10.1006/meth.2001.1262 11846609

[39] Bai X, Zhang Z, Wang J, Xu Z. Application of Transcriptome Sequencing Technology in Genetic Breeding of Livestock. Journal of Henan Agricultural Sciences. 2017; 46, 6–9.

[40] Wei H, Persson S, Mehta T, Srinivasasainagendra V, Chen L, Page GP, et al. Transcriptional Coordination of the Metabolic Network in Arabidopsis. Plant Physiology. 2006; 142:762–774. doi: 10.1104/pp.106.080358 16920875 PMC1586052

[41] Ng SK, Lau CC, Tan MP, Nor SAM, Danish-Daniel M, Afiqah‐Aleng N, et al. Using a transcriptomic approach to understand poor growth performance in farmed orange-spotted grouper (Epinephelus coioides) larvae: a case study in a commercial hatchery. New Zealand Journal of Marine and Freshwater Research. 2023; 1–20.

[42] Sun Y, Guo C, Wang D, Li XF, Xiao L, Zhang X, et al. Transcriptome analysis reveals the molecular mechanisms underlying growth superiority in a novel grouper hybrid (Epinephelus fuscogutatus♀ × E. lanceolatus♂). Bmc Genetics. 2016; 17, 24. 26785614 10.1186/s12863-016-0328-y PMC4719697

[43] Wang T, Wu X, Song L, Yang Y, Gong S, Zeng L, et al. Identification of candidate growth-related SNPs and genes using GWAS and transcriptome analyses in leopard coral grouper (Plectropomus leopardus). Aquaculture. 2023; 574:739677.

[44] Dana B-Z, Offir L, Levy AA, Barkai N. Hybrid vigor: The best of both parents, or a genomic clash? Current Opinion in Systems Biology. 2017; 6:22–27.

[45] Dong H, Nilsson L, Kurland CG. Gratuitous overexpression of genes in Escherichia coli leads to growth inhibition and ribosome destruction. Journal of Bacteriology. 1995; 177:1497–1504. doi: 10.1128/jb.177.6.1497-1504.1995 7883706 PMC176765

[46] Perfeito L, Ghozzi S, Berg J, Schnetz K, Lässig M. Nonlinear Fitness Landscape of a Molecular Pathway. PLoS Genetics. 2011; 7:e1002160. doi: 10.1371/journal.pgen.1002160 21814515 PMC3140986

[47] Meyer RC, Witucka-Wall H, Becher M, Blacha A, Boudichevskaia A, D-Rmann P, et al. Heterosis manifestation during early Arabidopsis seedling development is characterized by intermediate gene expression and enhanced metabolic activity in the hybrids. The Plant Journal. 2012; 71:669–683. doi: 10.1111/j.1365-313X.2012.05021.x 22487254

[48] Guo M, Zinselmeier C, Yang X, Crasta O, Smith BO, Bowen R, et al. Genome-wide transcript analysis of maize hybrids: allelic additive gene expression and yield heterosis. Theoretical & Applied Genetics. 2006; 113:831–845. 10.1007/s00122-006-0335-x 16868764

[49] Coolican SA, Samuel DS, Ewton DZ, Mcwade FJ, Florini JR. The Mitogenic and Myogenic Actions of Insulin-like Growth Factors Utilize Distinct Signaling Pathways. Journal of Biological Chemistry. 1997; 272:6653–6662. doi: 10.1074/jbc.272.10.6653 9045696

[50] Li J, Johnson SE. ERK2 is required for efficient terminal differentiation of skeletal myoblasts. Biochemical and Biophysical Research Communications. 2006; 345:1425–1433. doi: 10.1016/j.bbrc.2006.05.051 16729973

[51] Rommel C, Bodine SC, Clarke BA, Rossman R, Nunez L, Stitt TN, et al. Mediation of IGF-1-induced skeletal myotube hypertrophy by PI(3)K/Akt/mTOR and PI(3)K/Akt/GSK3 pathways. Nature Cell Biology. 2001; 3:1009–1013. doi: 10.1038/ncb1101-1009 11715022

[52] Engelman JA, Corcoran RB, Courtney KD. The PI3K pathway as drug target in human cancer. Journal of clinical oncology: official journal of the American Society of Clinical Oncology. 2010; 28:1075–1083. doi: 10.1200/JCO.2009.25.3641 20085938 PMC2834432

[53] Kritikou E. PTEN—a new guardian of the genome. Nature Reviews Molecular Cell Biology. 2007; 8:179.

