# Supplementary material for: Combining full-length transcriptome sequencing and next generation sequencing to provide insight into the growth superiority of the hybrid grouper (Cromileptes altivelas (♀) × Epinephelus lanceolatus (♂))
Source: PLoS One. 2024 Oct 9;19(10):e0308802. doi: 10.1371/journal.pone.0308802 (PMC11463768; doi:10.1371/journal.pone.0308802)
Supplement: S1 Table — (DOC) [file pone.0308802.s001.doc]

**S1 Table. The primers used for qRT-PCR**

| Primers | Sequences (5’-3‘) | Products (bp) |
| --- | --- | --- |
| AN32A | GTCATCTACGCCGTCTCCA | 247 |
|  | CCCCAACCTCACACATCTAA |  |
| G137B | TGAGTGAGTTTCTGGAGTGGTT | 196 |
|  | TGCGTTATTCAGTTTGACAGTT |  |
| PTEN | GGCAACAGTGTCAATACCTCG | 146 |
|  | CCCCAGACGGCAATAGAATA |  |
| ACTC | TCTTTCTCTCCGAGCCGC | 198 |
|  | CCTTCTGACCCATACCGACC |  |
| CAZA1 | GGAGAAGAATCAGGACCGC | 133 |
|  | CCTTATTTCCAATCCACCACA |  |
| FGFR3 | GTGTCTCCACCCACCGTCA | 187 |
|  | CAGAGTAGTGCATCTCAAAGCG |  |
| HSPB1 | GCCAAAGGGCTCTTCATC | 152 |
|  | CTGCTCTGGTGCCAATCA |  |
| MLRS | AGCCAAACCTCCACTGATACC | 159 |
|  | CCCAATGAAGCCAGCACG |  |
| MYSS | GCTGAGGTGTAACGGTGTGC | 141 |
|  | TTGTTGTCAATGAACTGTCCC |  |
| MYPC2 | AGCACAACAGACGAGCAAAC | 205 |
|  | TGAGTAAACTTAGGCACACAGG |  |
| RHOAB | TGCGTCCACTCTCCTATCC | 178 |
|  | CGGGTGTGCTCATCGTTA |  |
| STYL2 | CGGGGAGGAAAACACGGA | 186 |
|  | AAGGCGGAGAGGGGTATCG |  |
| TBA | CTCGCCACGGCAAATACA | 208 |
|  | CACACAGCCCTCTGGACCT |  |
| TBB1 | GACAACGAAGCCCTGTATGA | 153 |
|  | CAGTTTGCGGAGGTCAGC |  |
| TPM1 | TAGCCGCTCTCCCACTCT | 166 |
|  | CTGCTGCCTTCTTGTCTCC |  |
