# Supplementary material for: Combining full-length transcriptome sequencing and next generation sequencing to provide insight into the growth superiority of the hybrid grouper (Cromileptes altivelas (♀) × Epinephelus lanceolatus (♂))
Source: PLoS One. 2024 Oct 9;19(10):e0308802. doi: 10.1371/journal.pone.0308802 (PMC11463768; doi:10.1371/journal.pone.0308802)
Supplement: S2 Table — (DOC) [file pone.0308802.s002.doc]

**S2** Table. The results of the transcriptome integrity assessment based on BUSCO using aneukaryota (ODB9) core gene dataset

|  | Item | Number | Percent |
| --- | --- | --- | --- |
| Cal | Complete BUSCOs | 213 | 70.29% |
|  | Complete and single-copy BUSCOs | 179 | 59.07% |
|  | Complete and duplicated BUSCOs | 34 | 11.22% |
|  | Fragmented BUSCOs | 32 | 10.56% |
|  | Missing BUSCOs | 58 | 19.14% |
|  | Total BUSCO groups searched | 303 | 100.00% |
| Ela | Complete BUSCOs | 218 | 71.95% |
|  | Complete and single-copy BUSCOs | 183 | 60.40% |
|  | Complete and duplicated BUSCOs | 35 | 11.55% |
|  | Fragmented BUSCOs | 25 | 8.25% |
|  | Missing BUSCOs | 60 | 19.80% |
|  | Total BUSCO groups searched | 303 | 100.00% |
| Hyb | Complete BUSCOs | 229 | 75.58% |
|  | Complete and single-copy BUSCOs | 151 | 49.83% |
|  | Complete and duplicated BUSCOs | 78 | 25.74% |
|  | Fragmented BUSCOs | 30 | 9.90% |
|  | Missing BUSCOs | 44 | 14.52% |
|  | Total BUSCO groups searched | 303 | 100.00% |
