# Supplementary material for: Combining full-length transcriptome sequencing and next generation sequencing to provide insight into the growth superiority of the hybrid grouper (Cromileptes altivelas (♀) × Epinephelus lanceolatus (♂))
Source: PLoS One. 2024 Oct 9;19(10):e0308802. doi: 10.1371/journal.pone.0308802 (PMC11463768; doi:10.1371/journal.pone.0308802)
Supplement: S3 Table — (DOC) [file pone.0308802.s003.doc]

**S3 Table. BLAST analysis of the full-length transcripts against public databases**

| Item | Count | Percentage |
| --- | --- | --- |
| All | 204,322 |  |
| Annotation | 125,305 | 61.33% |
| Swiss-prot | 53,314 | 26.09% |
| Pfam | 69,506 | 34.02% |
| COG | 16,541 | 8.10% |
| GO | 96,729 | 47.34% |
| KEGG | 93,622 | 45.82% |
| KOG | 67,478 | 33.03% |
| eggNOG | 96,458 | 47.21% |
| Nr | 120,463 | 58.96% |
