# Supplementary material for: Combining full-length transcriptome sequencing and next generation sequencing to provide insight into the growth superiority of the hybrid grouper (Cromileptes altivelas (♀) × Epinephelus lanceolatus (♂))
Source: PLoS One. 2024 Oct 9;19(10):e0308802. doi: 10.1371/journal.pone.0308802 (PMC11463768; doi:10.1371/journal.pone.0308802)
Supplement: S4 Table — (DOC) [file pone.0308802.s004.doc]

**S4 Table.** **Summary of the alignment between next-generation sequencing reads and PacBio transcripts**

| Sample | Total reads | Uniquely mapped reads (%) | Reads mapped to multiple loci (%) | Reads mapped to too many loci (%) | Unmapped reads (%) |
| --- | --- | --- | --- | --- | --- |
| lsb-b-1 | 21,217,451 | 48.69% | 35.84% | 2.95% | 12.52% |
| lsb-b-2 | 21,599,185 | 46.48% | 35.65% | 4.25% | 13.62% |
| lsb-b-3 | 20,725,002 | 46.18% | 38.21% | 4.19% | 11.42% |
| lsb-b-4 | 20,648,666 | 48.77% | 33.66% | 1.79% | 15.78% |
| lsb-b-5 | 20,924,672 | 43.14% | 38.63% | 5.77% | 12.46% |
| lsb-l-1 | 20,798,982 | 35.09% | 40.06% | 12.87% | 11.98% |
| lsb-l-2 | 20,932,047 | 34.41% | 40.24% | 13.48% | 11.87% |
| lsb-l-3 | 19,500,556 | 35.45% | 43.05% | 11.04% | 10.46% |
| lsb-l-4 | 20,944,758 | 37.24% | 43.02% | 10.77% | 8.97% |
| lsb-l-5 | 20,464,641 | 35.75% | 39.84% | 12.30% | 12.11% |
| lsb-m-1 | 23,876,341 | 25.99% | 36.46% | 25.02% | 12.53% |
| lsb-m-2 | 20,959,599 | 27.51% | 36.72% | 23.15% | 12.62% |
| lsb-m-3 | 21,601,911 | 26.04% | 35.30% | 22.93% | 15.73% |
| lsb-m-4 | 20,953,870 | 27.34% | 34.62% | 22.45% | 15.59% |
| lsb-m-5 | 20,706,367 | 24.80% | 36.32% | 22.35% | 16.53% |
| ld-b-1 | 21,533,894 | 47.25% | 38.91% | 1.33% | 12.51% |
| ld-b-2 | 21,640,389 | 46.34% | 38.13% | 1.43% | 14.10% |
| ld-b-3 | 21,471,823 | 46.14% | 38.02% | 1.45% | 14.39% |
| ld-b-4 | 20,023,574 | 49.07% | 36.21% | 1.71% | 13.01% |
| ld-b-5 | 21,251,960 | 45.53% | 35.42% | 5.35% | 13.70% |
| ld-l-1 | 22,224,522 | 38.49% | 38.34% | 10.68% | 12.49% |
| ld-l-2 | 21,359,938 | 43.11% | 36.49% | 9.15% | 11.25% |
| ld-l-3 | 20,674,906 | 34.98% | 43.39% | 14.21% | 7.42% |
| ld-l-4 | 21,216,080 | 42.41% | 35.15% | 8.48% | 13.96% |
| ld-l-5 | 19,823,336 | 36.42% | 39.17% | 14.26% | 10.15% |
| ld-m-1 | 20,361,534 | 22.03% | 42.61% | 24.52% | 10.84% |
| ld-m-2 | 20,819,843 | 22.84% | 40.22% | 27.56% | 9.38% |
| ld-m-3 | 20,858,075 | 24.56% | 44.56% | 21.32% | 9.56% |
| ld-m-4 | 21,335,821 | 23.25% | 41.42% | 22.69% | 12.64% |
| ld-m-5 | 21,533,893 | 22.25% | 44.71% | 24.03% | 9.01% |
| hlb-b-1 | 19,234,300 | 46.24% | 37.97% | 4.03% | 11.76% |
| hlb-b-2 | 20,857,484 | 44.54% | 35.06% | 2.99% | 17.41% |
| hlb-b-3 | 21,254,265 | 42.16% | 35.88% | 7.75% | 14.21% |
| hlb-b-4 | 20,876,060 | 46.24% | 37.26% | 3.61% | 12.89% |
| hlb-b-5 | 21,560,551 | 44.28% | 37.73% | 4.76% | 13.23% |
| hlb-l-1 | 22,054,143 | 34.93% | 41.34% | 14.09% | 9.64% |
| hlb-l-2 | 21,409,218 | 32.52% | 40.88% | 13.88% | 12.72% |
| hlb-l-3 | 20,437,498 | 33.84% | 40.46% | 15.80% | 9.90% |
| hlb-l-4 | 21,824,180 | 36.79% | 40.38% | 12.63% | 10.20% |
| hlb-l-5 | 21,187,594 | 30.37% | 43.39% | 16.83% | 9.41% |
| hlb-m-1 | 22,980,390 | 26.35% | 41.59% | 21.91% | 10.15% |
| hlb-m-2 | 21,661,276 | 24.11% | 42.41% | 23.08% | 10.40% |
| hlb-m-3 | 22,133,248 | 25.82% | 38.04% | 23.52% | 12.62% |
| hlb-m-4 | 22,295,929 | 23.67% | 38.36% | 26.82% | 11.15% |
| hlb-m-5 | 22,044,533 | 22.66% | 41.66% | 25.83% | 9.85% |

Note: lsd-b, lsd-l, lsd-m represented the brain, liver and muscle tissue in Cal; ld-b, ld-l, ld-m represented the brain, liver and muscle tissue in Ela; hlb-b, hlb-l, hlb-m represented the brain, liver and muscle tissue in Hyb.
