# Supplementary material for: Combining full-length transcriptome sequencing and next generation sequencing to provide insight into the growth superiority of the hybrid grouper (Cromileptes altivelas (♀) × Epinephelus lanceolatus (♂))
Source: PLoS One. 2024 Oct 9;19(10):e0308802. doi: 10.1371/journal.pone.0308802 (PMC11463768; doi:10.1371/journal.pone.0308802)
Supplement: S5 Table — (DOC) [file pone.0308802.s005.doc]

**S5** Table. Gene annotation of DEGs in the growth-related correlation network

| Tissue | Gene ID | Nr annotation | SYMBOL | logFC(Hyb vs Cal) | logFC(Hyb vs Ela) |
| --- | --- | --- | --- | --- | --- |
| brain | lsb_transcript_40720 | acidic leucine-rich nuclear phosphoprotein 32 family member A | AN32A | 0.78 | -2.74 |
|  | hlb_transcript_85540 | integral membrane protein GPR137B | G137B | 0.93 | -2.95 |
|  | lsb_transcript_51125 | Phosphatidylinositol 3,4,5-trisphosphate 3-phosphatase and dual-specificity protein phosphatase | PTEN | 1.17 | -4.28 |
| muscle | lsb_transcript_23706 | actin, cytoplasmic 2 isoform X1 | ACTC | 1.26 | -4.90 |
|  | lsb_transcript_36759 | F-actin-capping protein subunit alpha-1b | CAZA1 | 0.82 | -4.60 |
|  | lsb_transcript_41764 | Titin | FGFR3 | 0.63 | -10.14 |
|  | lsb_transcript_15674 | heat shock protein beta-8 | HSPB1 | 0.82 | -8.73 |
|  | lsb_transcript_43254 | myosin light chain 2 | MLRS | 0.44 | -5.37 |
|  | lsb_transcript_46190 | myosin heavy chain | MYSS | 0.31 | -6.60 |
|  | lsb_transcript_58205 | myosin binding protein Cb isoform X1 | MYPC2 | 1.48 | -7.41 |
|  | hlb_transcript_19441 | rho-related GTP-binding protein RhoB | RHOAB | 0.42 | -6.61 |
|  | hlb_transcript_94027 | inactive dual specificity phosphatase 27 isoform X2 | STYL2 | 0.64 | -7.03 |
|  | lsb_transcript_77240 | tubulin alpha chain-like | TBA | 0.66 | -4.95 |
|  | hlb_transcript_6965 | tubulin beta-1 chain | TBB1 | 0.31 | -4.55 |
|  | hlb_transcript_130185 | tropomyosin alpha-1 chain-like isoform X5 | TPM1 | 1.19 | -6.37 |
